# Supplementary material for: Geographic and demographic heterogeneity of SARS-CoV-2 diagnostic testing in Illinois, USA, March to December 2020
Source: BMC Public Health. 2021 Jun 10;21:1105. doi: 10.1186/s12889-021-11177-x (PMC8189821; doi:10.1186/s12889-021-11177-x)
Supplement: Supplementary file 1 — Additional file 1: Figure S1. Calculation of fdeathdet from Illinois COVID-19-attributed deaths and excess select cause deaths. Figure S2. Testing rate per capita by race/ethnicity and COVID-19 Region. Population denominators were drawn from ACS 2018. “Native” included both “American Indian or Alaskan Native” and “Native Hawaiian or Other Pacific Islander”. Native populations were excluded from Figures 6 and 8 due to small population denominators after stratifying by age. The small denominators are reflected in the large fluctuations in the Native timeseries in this figure. Figure S3. Daily COVID-19 hospital census by COVID-19 Region (subplots) and super-region (colors) in 2020. Lines show 7-day rolling averages. Data for med/surg occupancy are not available prior to May 2020. Med/surg census covers all admitted patients who are not in the intensive care unit (ICU). Figure S4. Sensitivity of CFR to case race/ethnicity assignment. Alternate scenarios were run in which cases with “unknown” ethnicity were: 1) Assumed to be non-Hispanic and assigned to the recorded racial group (solid line, and identical to Figure 8), 2) Assigned to the “unknown” racial group (dashed line), or 3) Assumed to be Hispanic (dotted line). For Hispanic-Latino, scenarios #1 and #2 are identical. For Black, White, and Asian, scenarios #2 and #3 are identical. Assumption of non-Hispanic ethnicity slightly, but significantly, decreases CFR for older non-Hispanic White and Black populations. Table S1. Total hospital beds by COVID-19 Region. Bed availability data from September 1, 2020 via IDPH website. Beds available for COVID refers to number of beds not occupied by non-COVID patients. [file 12889_2021_11177_MOESM1_ESM.docx]

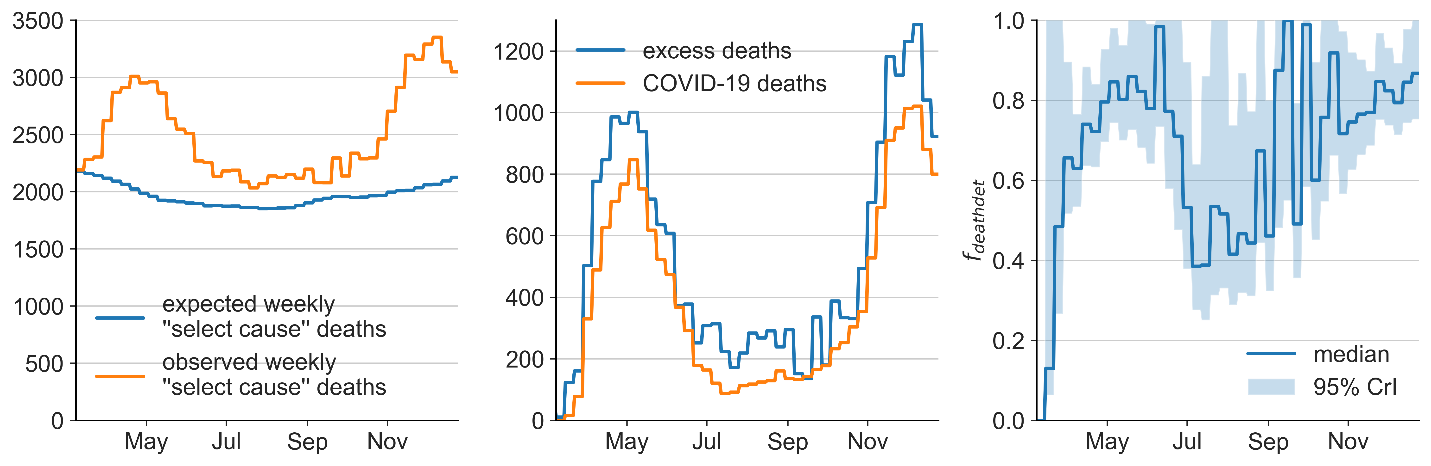


Figure S1. Calculation of *f_deathdet_* from Illinois COVID-19-attributed deaths and excess select cause deaths.


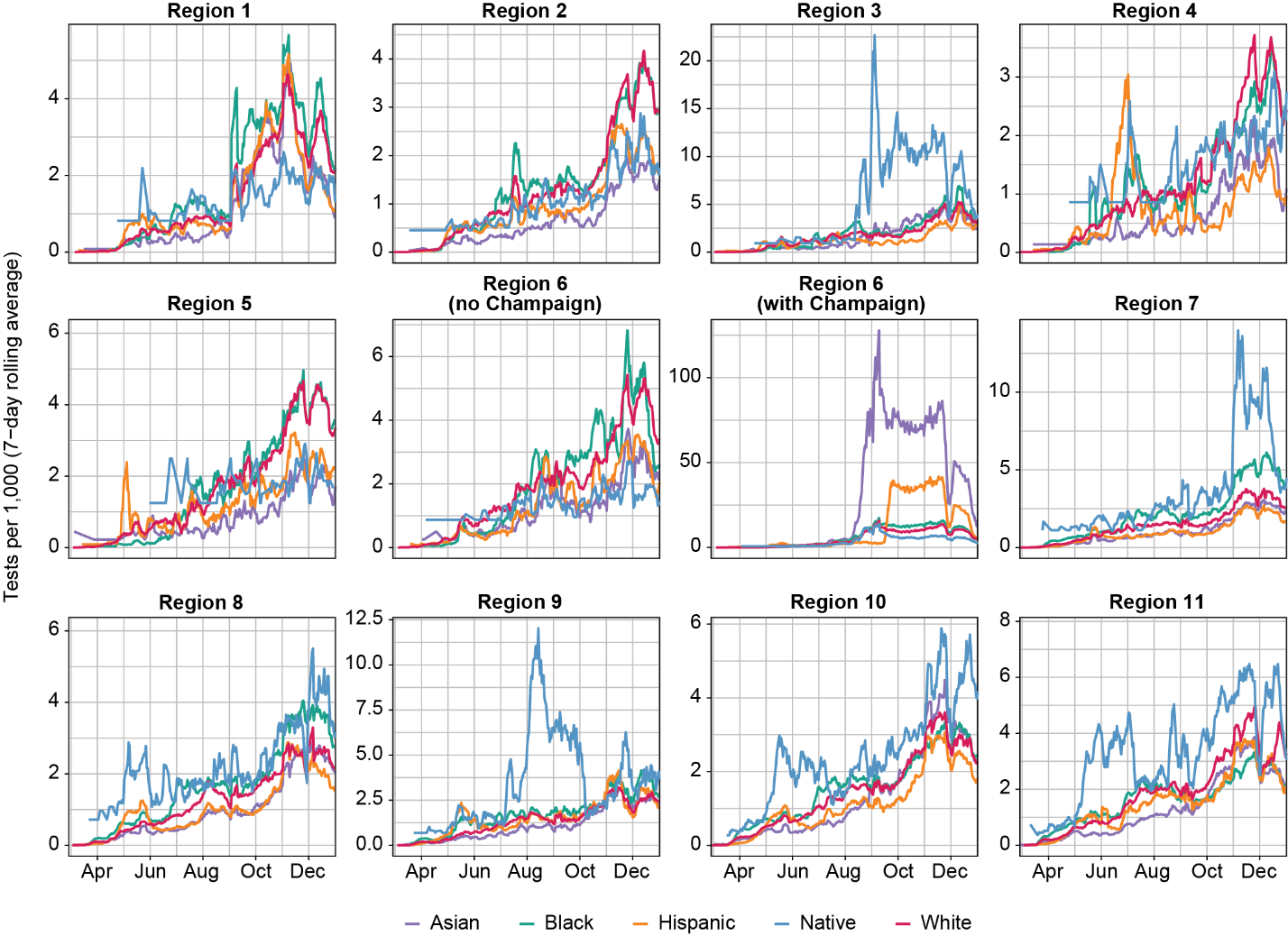


Figure S2. Testing rate per capita by race/ethnicity and COVID-19 Region. Population denominators were drawn from ACS 2018. “Native” included both “American Indian or Alaskan Native” and “Native Hawaiian or Other Pacific Islander”. Native populations were excluded from Figures 6 and 8 due to small population denominators after stratifying by age. The small denominators are reflected in the large fluctuations in the Native timeseries in this figure.


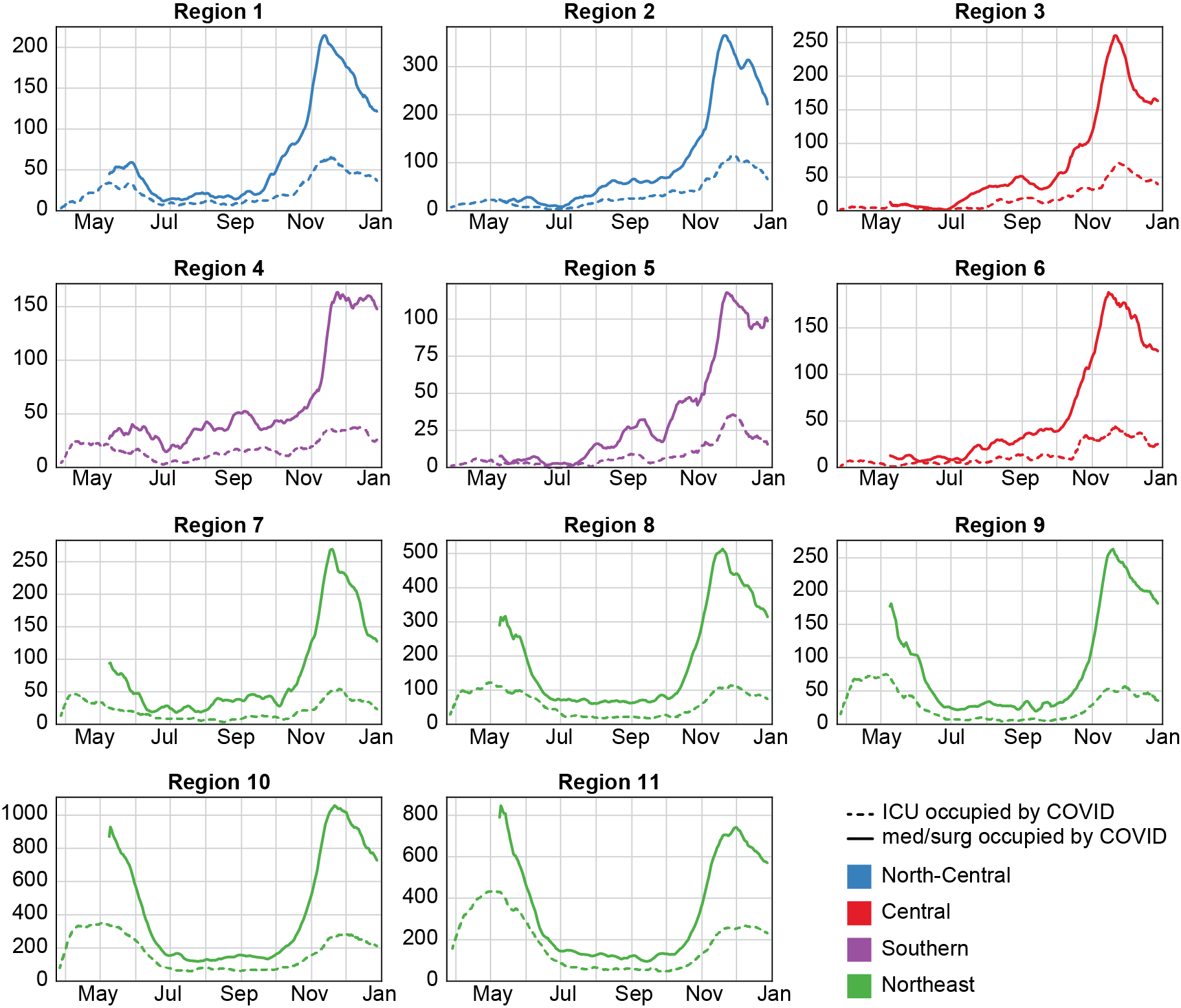


Figure S3. Daily COVID-19 hospital census by COVID-19 Region (subplots) and super-region (colors) in 2020. Lines show 7-day rolling averages. Data for med/surg occupancy are not available prior to May 2020. Med/surg census covers all admitted patients who are not in the intensive care unit (ICU).


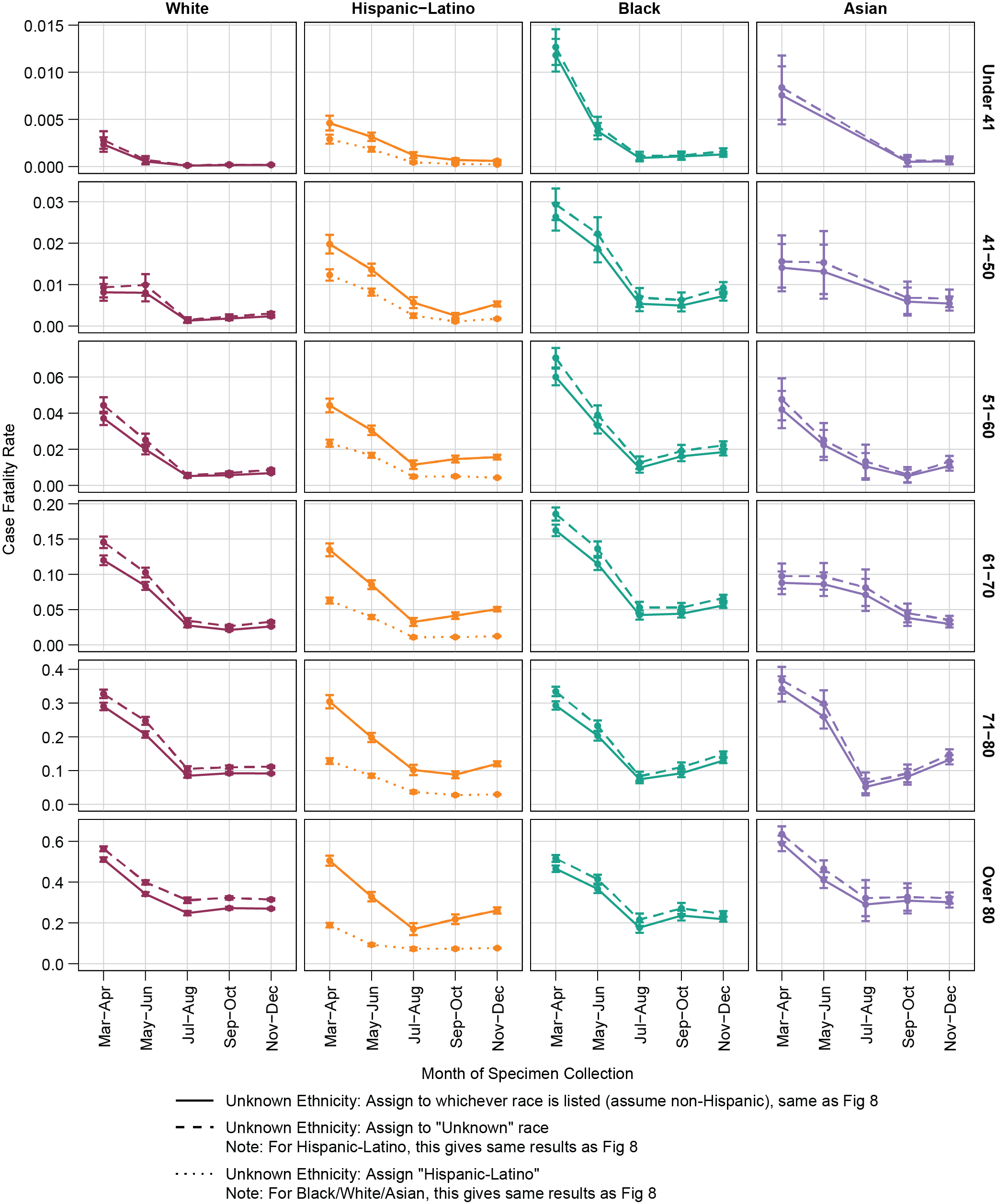


Figure S4. Sensitivity of CFR to case race/ethnicity assignment. Alternate scenarios were run in which cases with “unknown” ethnicity were: 1) Assumed to be non-Hispanic and assigned to the recorded racial group (solid line, and identical to Figure 8), 2) Assigned to the “unknown” racial group (dashed line), or 3) Assumed to be Hispanic (dotted line). For Hispanic-Latino, scenarios #1 and #2 are identical. For Black, White, and Asian, scenarios #2 and #3 are identical. Assumption of non-Hispanic ethnicity slightly, but significantly, decreases CFR for older non-Hispanic White and Black populations.

Table S1. Total hospital beds by COVID-19 Region. Bed availability data from September 1, 2020 via IDPH website. Beds available for COVID refers to number of beds not occupied by non-COVID patients.

| COVID-19 Region | population | Med/Surg total beds | ICU total beds | Med/Surg beds available for COVID | ICU beds available for COVID | Med/Surg beds available for COVID per 10,000 | ICU beds available for COVID per 10,000 |
| --- | --- | --- | --- | --- | --- | --- | --- |
| 1 | 660965 | 939 | 229 | 428 | 124 | 6.48 | 1.88 |
| 2 | 1243906 | 1876 | 323 | 870 | 156 | 6.99 | 1.25 |
| 3 | 556776 | 1141 | 150 | 475 | 73 | 8.53 | 1.31 |
| 4 | 656946 | 862 | 130 | 343 | 86 | 5.22 | 1.31 |
| 5 | 403659 | 668 | 99 | 370 | 65 | 9.17 | 1.61 |
| 6 | 739098 | 1087 | 178 | 461 | 112 | 6.24 | 1.52 |
| 7 | 800605 | 820 | 174 | 320 | 63 | 4.00 | 0.79 |
| 8 | 1455324 | 1906 | 431 | 761 | 222 | 5.23 | 1.53 |
| 9 | 1004309 | 1117 | 254 | 513 | 140 | 5.11 | 1.39 |
| 10 | 2693959 | 3680 | 772 | 1237 | 354 | 4.59 | 1.31 |
| 11 | 2456274 | 4475 | 1066 | 1526 | 516 | 6.21 | 2.10 |
